# Toward MR protocol-agnostic, bias-corrected brain age predicted from clinical-grade MRIs

**DOI:** 10.21203/rs.3.rs-3229072/v1

**Published:** 2023-08-11

**Authors:** Pedro Valdes-Hernandez, Chavier Laffitte Nodarse, Julio Peraza, James Cole, Yenisel Cruz-Almeida

**Affiliations:** University of Florida; University of Florida; Florida International University; University College London; University of Florida

**Keywords:** Clinical Multimodal MRI, Synthetic MPRAGE, brain-PAD, brain age gap, DeepBrainNet, transfer learning, research-grade MRI

## Abstract

The predicted brain age minus the chronological age (‘brain-PAD’) could become a clinical biomarker. However, most brain age methods were developed to use research-grade high-resolution T1-weighted MRIs, limiting their applicability to clinical-grade MRIs from multiple protocols. To overcome this, we adopted a double transfer learning approach to develop a brain age model agnostic to modality, resolution, or slice orientation. Using 6,224 clinical MRIs among 7 modalities, scanned from 1,540 patients using 8 scanners among 15 + facilities of the University of Florida’s Health System, we retrained a convolutional neural network (CNN) to predict brain age from synthetic research-grade magnetization-prepared rapid gradient-echo MRIs (MPRAGEs) generated by a deep learning-trained ‘super-resolution’ method. We also modeled the “regression dilution bias”, a typical overestimation of younger ages and underestimation of older ages, which correction is paramount for personalized brain age-based biomarkers. This bias was independent of modality or scanner and generalizable to new samples, allowing us to add a bias-correction layer to the CNN. The mean absolute error in test samples was 4.67–6.47 years across modalities, with similar accuracy between original MPRAGEs and their synthetic counterparts. Brain-PAD was also reliable across modalities. We demonstrate the feasibility of clinical-grade brain age predictions, contributing to personalized medicine.

## Introduction

Brain age predicted from brain Magnetic Resonance Images (MRIs) using machine learning methods^1-3^ has the potential to be a biomarker of disease^4-6^. The difference between the predicted brain age and the chronological age, namely, the ‘brain-PAD’ or ‘brain age gap’, has been shown to be sensitive to pathologies or good lifestyle factors. People with higher brain-PAD values are more likely to have a disease or be at risk of developing a disease; whereas people with lower brain-PAD values are more likely to be healthier^2,4,13,5-12^.

Most brain age prediction methods were trained with, and are thus better suitable for, high-resolution (with near isotropic 1mm resolution) three-dimensional (3D) brain T1-weighted (T1w) MRIs primarily obtained for research purposes, such as the magnetization-prepared rapid gradient-echo (MPRAGE) or the spoiled gradient recalled (3D-SPGR) sequences. This limits the clinical applicability of these methods since the models trained on high-resolution high-quality research scans may not generalize well to the typical brain MRIs data sequences acquired at scale in a myriad of hospital across the world. For example, a clinical brain MR scanning session could only include a fast two-dimensional (2D) T1w, or non-T1 modalities like the T2-weighted (T2w) or the Fluid Attenuation Inversion recovery (FLAIR), with good axial, coronal or sagittal in-plane resolution but poor slice resolution (e.g., thickness ~5-10 mm). Therefore, models that can accurately predict brain age from MRIs acquired using any clinical MR protocol (i.e., clinical-grade MRIs) are needed.

There have been some efforts to deal with non-T1w modalities in the literature. Cole (2020)^14^ used six modalities as features to train a brain age model in 2205 participants of the UK Biobank. With the same goal, Rokicki et al. (2021)^6^ used several T1w-based morphometric features and the T1w/T2w ratio in 750 healthy participants and Millar et al (2023)^15^ used the structural and functional MRIs of 390 healthy participants. However, the former study advocates for the use of all modalities combined as features for brain age prediction, while the latter studies were trained with relatively small sample sizes. Moreover, both studies used research-grade MRI databases. An exception of the use of research-grade MRIs is Wood et al. (2022)^16^ who used clinical-grade T2 weighted (T2w) and diffusion-weighted images (DWI) were used to train a convolutional neural network (CNN) to predict brain age. However, the technique is restricted to axial MRIs of these two modalities.

Here, we explore an alternative approach leveraging existing brain aging methods to develop a brain age prediction method agnostic to modality, resolution, or slice orientation. To achieve that, we advocate for the transfer of what super-resolution (SR) methods learned about the relationship between clinical-grade and research-grade MRIs. In particular, we incorporate a preprocessing step before brain age prediction that is the prediction of a research-grade 1-mm isotropic MPRAGE from the clinical MRI using the “Synthetic SR” (SynthSR)^17^. SynthSR was trained using a concatenation of a U-Net regression and a U-net segmentation on a dataset of 1-mm isotropic 3D MPRAGE scans and companion neuroanatomical labels. It predicts a synthetic MPRAGE from an input neuroimage of any modality, orientation, or resolution with enough quality to produce morphometric results similar to those obtained when using the actual MPRAGE, and it is publicly available in FreeSurfer.

To predict brain age from either the original or synthetic MPRAGEs, we repurposed DeepBrainNet (i.e., a publicly available CNN trained on a large and heterogeneous research-grade T1 w dataset) via transfer learning retraining on the synthetic MPRAGEs predicted from 6,224 clinical MRIs, distributed among 7 different modalities and 8 different scanners, from 1,540 patients that were scanned at more than 15 facilities of the University of Florida (UF) Health System. With this double transfer learning approach, we meet midway between leveraging what SynthSR and DeepBrainNet have learned, making the latter potentially agnostic to the modality, resolution, or clinical nature of the MRI. We thus hypothesized that the predictive accuracy of our new approach applied to clinical-grade MRIs from any MR protocol would be comparable to the accuracy when using the DeepBrainNet on actual research-grade MPRAGEs.

When predicting brain age in new samples, a bias consisting of an overestimation of younger ages and an underestimation of older ages greatly reduces accuracy. This is the so-called “regression dilution” due to errors in the features (i.e., the MRIs) used to predict the outcome (i.e., brain age). As long as the bias is linear and not severe, it can be accounted for by regressing out chronological age from brain-PAD^18^. But a reliable linear regression requires a minimum sample size for the new data, rendering the resulting brain-PAD invalid as a personalized biomarker of disease. Thus, generalizable models of the bias are needed. Here, we set out to characterize the bias in brain age in our clinical-grade MRI data. We hypothesized that the bias can be generalized to newer samples, thus allowing the correction of individual brain-PADs. We also hypothesized that this bias would differ by modality and/or scanner model and thus, accounting for these interactions would yield better accuracy in newer samples. Finally, we hypothesized that the corrected brain-PAD would be consistent across modalities and repeat scans within subjects.

## Results

### Sample distribution across modalities and scanners

**Figure 1:**
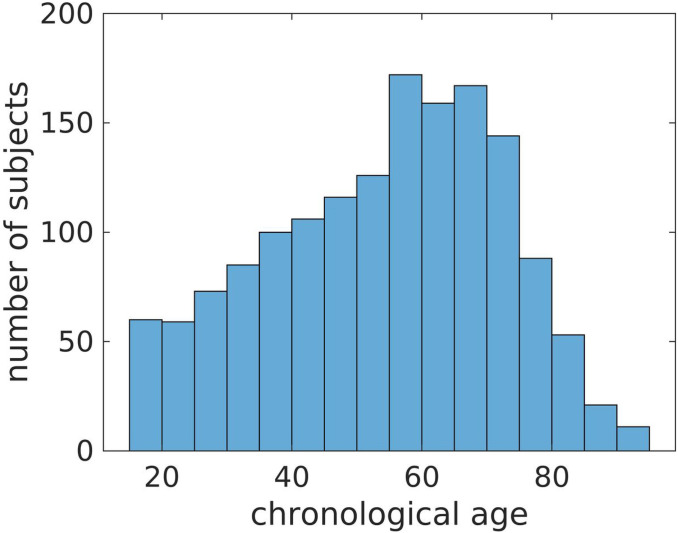
Distribution of chronological ages in the sample.

This study used MRIs from a randomly selected initial sample of subjects that were scanned between February 2017 and March 2021 at more than 15 facilities of the UF Health System. We received the raw DICOMS from 24,732 MRIs of 1,727 patients. After removing all non-brain and partial-brain MRIs, we ended up with a total of 7,005 brain MRIs distributed among 8 modalities and repetitions. After quality control (QC) and synthetic MPRAGE prediction, we eliminated 781 MRIs to obtain a final sample of 6,224 whole-brain MRIs across 7 modalities from 1,540 patients. Table 1 shows how these MRIs are distributed among the 7 modalities, scanner models and repetitions. The modalities were the 3D MPRAGE, and the 2D MRIs: T1w and T2-weighted (T2w), T1w and T2w Fluid attenuated inversion recovery (FLAIR), T1w and T2w Gradient Echo (GRE) and Inversion Recovery (IR). **Table S1** in the Supplemental Materials evidences the high variability in some of the MRI parameters and slice orientations in the sample. The sample had 1,039 females and 501 males with chronological age ranging from 15 to 95 years, with a mean, median, and standard deviation of 53.5, 56 and 18 years (see Fig. 1).

### Predicted MPRAGEs

**Figure 2:**
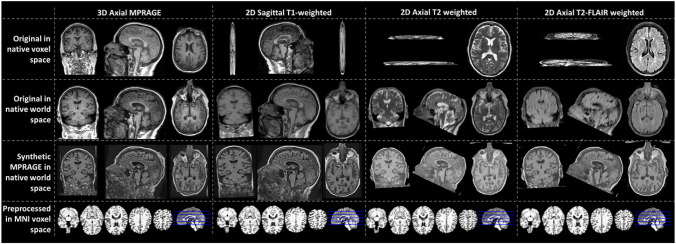
Predicted MPRAGEs for three of the five modalities included in this study. The data belongs to a single participant. The top row shows selected slices in the slice, phase-encoding and readout directions (left, right top and right bottom, respectively). The bottom row shows the bottommost (z = −27 mm, slice 46), topmost (z = 53 mm, slice 126), and three intermediate slices (z = −7, 13, and 33mm, slices 66, 86 and 106, respectively) of the 80 slices used for brain age prediction. To show images in world space, trilinear interpolation was used. FLAIR: Fluid attenuated inversion recovery.

Figure 2 shows SynthSR in action for some of the MRI modalities of a participant of the sample. In the figure, the first two rows expose the poor resolution of clinical-grade MRIs along the slice direction. The third row shows how SynthSR remedies this, and the fourth shows the final preprocessed MRI, in the Montreal Neurological Institute (MNI) space, required by DeepBrainNet to predict brain age.

### Uncorrected brain age predictions

A DeepBrainNet model consists of a two-dimensional (2D) CNN connected to a dense layer with 1024 units with a Rectified Linear Unit (ReLU) activation, an 80% dropout layer (when training), and a single output layer with linear activation (the predicted brain age)^2^. In the original paper^2^, DeepBrainNet models were trained with research-grade MRIs to predict brain age independently from 80 selected slices of an MRI in the MNI space. The whole brain age is then calculated as the median across slices.

In this paper, we instead used the synthetic MPRAGEs predicted from the clinical-grade MRIs of 77% of the participants of our sample (the training set) to retrain two CNNs of the DeepBrainNet model: the “InceptionResNetV2”^19^ and the “Visual Geometry Group” network with 16 layers (VGG16)^20^. This retraining was also performed under several configurations of hyper-parameters. We then selected the CNN and hyper-parameter configuration yielding the lowest MAE of the brain age predictions via three-fold cross-validation. Also, in the non-training fold of each iteration of the cross-validation (33% of the participants of the training set), a subset of the participants was reserved for fitting the linear model characterizing the above-mentioned regression dilution bias (results associated with this step are presented in the next section). The training set was defined by the participants exclusively having the only four modalities with enough samples to provide robust training accuracy, i.e., the original MPRAGEs, as well as the synthetic MPRAGEs, T1ws, T2ws, and T2wFLAIRs (see Table 1). The remaining 23% of the participants, that could also have other modalities, were reserved for other purposes detailed below.

The configuration that minimized the bias-uncorrected MAE, averaged across folds, included the VGG16 model, with a batch size of 160 slices, learning rate of 7e-5 and no weights in the observations based on the frequency of their ages in the sample. In a first stage of each cross-validation iteration and hyper-parameter configuration, we loaded the original DeepBrainNet model, set the weights of the fully connected and output layers as trainable, while the weights of the rest of the network were frozen, and trained the model using a maximum of 20 epochs. In most of the iterations the algorithm stopped between the 15th and 20th epoch due to early stopping criteria defined to avoid overfitting. In a second stage of the iteration, we unfroze all the layers of the model and re-trained it with another maximum of 20 epochs. Notably, the algorithm stopped just after the first, second or third epoch due to overfitting, indicating that the retraining was mostly needed in the upper layers. Finally, the selected model and hyper-parameter configuration were used to train a final model on 90% of the training set, dedicating 10% to monitor overfitting (i.e., 70% and 7% of the whole set of participants, respectively).

**Figure 3:**
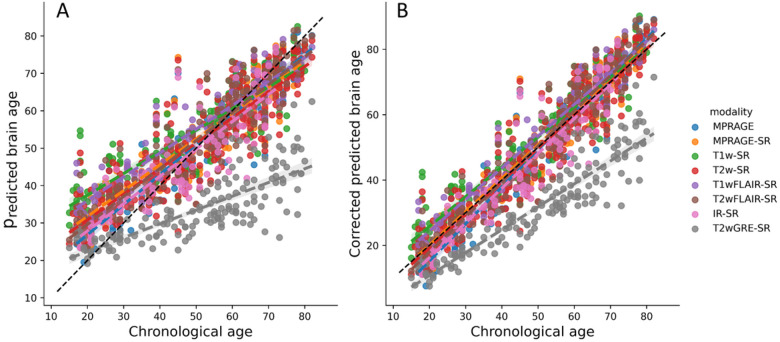
Brain age prediction using the selected retrained CNN (VGG16-based DeepBrainNet) for the MRIs of the testing held-out sample. A) Uncorrected and B) bias corrected. The colored lines represent the slope of the linear relation between the chronological and the predicted brain age for each modality. MPRAGE: Magnetization-prepared rapid gradient-echo. T1w: T1-weighted. T2w: T2-weighted. FLAIR: Fluid attenuated inversion recovery. GRE: Gradient Echo. IR: Inversion Recovery. [Modality]-SR: Super-resolution synthetic MPRAGE version of [modality].

The final model was tested in part of the above-mentioned 23% of the participants, specifically in 14.5% of the of participants (the testing set). This set was conveniently defined by the participants not only having MRIs of the modalities used for training but also having other modalities with sample sizes so small that they were not suitable to be part of the training set, i.e., T1wFLAIR, IR, and T2wGRE (see Table 1). This allowed for the evaluation of the generalization error not only in new participants but also in new modalities “unseen” by the retrained model. Figure 3A shows the brain age predictions using the selected retrained DeepBrainNet model in the testing set. The bias in the predictions, that overestimate younger ages and underestimates older ages, is visibly exposed by the slope of the linear relation between the chronological and predicted brain ages. Table 2 summarizes the measures of accuracy, including the MAE. These results also show that the model greatly underperforms for one of the modalities not used for training, i.e., the T2wGRE. Notably, this modality was obtained at a single facility among the more than 15 facilities of the UFHealth System, and with the Signa HDxt GE Medical Systems 1.5 T scanner.

### Corrected brain age predictions

As mentioned above, each cross-validation iteration involved two folds for training (66% of the participants in the training set). The third fold was split into 23% (to avoid confusion, we clarify that this proportion is by coincidence the same as the abovementioned proportion that is not part of the training set) of the participants of the training set for monitoring overfitting, and the reserved 10% to characterize the linear bias during cross-validation. This characterization was done by fitting the linear correction model: brainage∼prediction (in Wilkinson’s notation), where *prediction* was one among several linear models that could include interactions with modality or scanner model. After applying the correction correctedbrainage=chronologicalage+brainage−prediction^18^ to the brain age predictions of the retrained DeepBrainNet models, we selected the linear model that minimized the corrected MAE in the evaluation set, averaged across folds, for the above-selected CNN and hyper-parameter configuration. This linear model was brainage∼chronologicalage, thus rejecting our hypothesis that considering a linear model of the bias dependent of modality and/or scanner type would result in a lower generalization error.

Finally, we estimated the final parameters of the selected linear model using the remaining part of the 23% that was not part of the training set and testing set, i.e., 23%−14.5%=8.5% (the linear correction set), specifically held for this purpose. The intercept and slope of the linear correction model were 15.2 years and 0.7, respectively. This linear model was added as an additional layer, which takes both the predicted brain age and the chronological age as inputs, to the selected retrained DeepBrainNet model’s architecture for the deployment of a corrected retrained DeepBrainNet model.

Figure 3B shows the brain age predictions using the selected retrained and corrected DeepBrainNet model in the testing set, and Table 3 summarizes the measures of accuracy. Both items provide the results needed to draw conclusions regarding our hypothesis: that our new, bias-corrected model applied to synthetic MPRAGEs predicted from clinical-grade brain MRIs of any modality can be generalized to newer samples with an accuracy comparable to that obtained when using the DeepBrainNet model on actual research-grade MPRAGEs.

### Internal consistency of brain age predictions across modalities and repetitions

**Figure 4:**
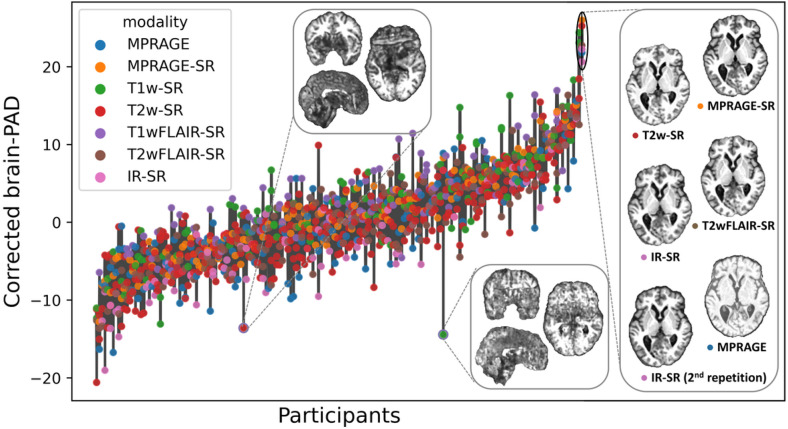
Variability of the corrected predicted corrected brain-PADs for each participant (ordered from the lowest to the highest brain-PAD). The bars represent the range of PAD values within each subject. To illustrate the possible causes of some of the outliers, we show the synthetic MPRAGEs of the two images with corrected brain-PAD farthest from the mean in their own within-subject group. These images, of very poor quality, were not detected by the QC described in this study. The inset to the right also shows the original MPRAGE and Synthetic MPRAGEs for the subject having the highest average corrected brain-PAD (26.7 years) in the test set (this participant had no T1wFLAIR or T1w but had two IRs).

To test our second hypothesis, that the corrected brain-PAD would be consistent across modalities and repeat scans within subjects, we assessed the intra-subject reliability of the predictions. First, we evaluated the Cronbach’s alpha, a measure of internal consistency^21^, on the brain-PAD. Cronbach’s alpha of the corrected brain-PADs was 0.92 with 95% CI of [0.90, 0.93] for the whole testing set and 0.95 with 95% CI of [0.94, 0.96] when excluding the T2wGRE. We also calculated the mean absolute difference between the corrected brain-PADs and their average for each subject. The average of this measure across subjects was 4.8 years with a bootstrapped 95% CI of [4.5, 5.1] years for the whole testing set, 2.4 years with a bootstrapped 95% CI of [2.2, 2.5] years when excluding the T2wGRE MRIs, and 2.0 years with a bootstrapped 95% CI of [1.9, 2.2] when excluding all modalities that were not used to re-train the model. Figure 4 depicts the predictions for each participant.

## Discussion

In this paper, we have demonstrated the feasibility of using clinical MRIs of any modality for brain aging prediction based on convolutional neural networks, the VGG16-based DeepBrainNet in particular. Our main finding is that, by leveraging the relationship between clinical MRIs and high-resolution research-grade MPRAGEs learned by “synthSR”, it seems now possible to predict brain ages from clinical-grade MRIs of any modality, in-plane resolution or slice thickness or orientation.

As discussed by de Lange and Cole (2020)^18^, the linear bias provoked by the so-called “regression dilution” tends to overestimate younger predicted ages and overestimate older ones. This linear bias does not have to be characterized or removed from brain age predictions prior to performing group analysis using brain-PAD as the dependent variable, since chronological age can be directly used as a covariate. However, the characterization of the bias is necessary when brain age is predicted for single-case or smaller MRI samples, which is the cornerstone of future brain age-based biomarkers in personalized medicine. Our second finding is that this linear bias in the brain age prediction can be successfully removed in newer participants.

Our third finding is that our brain age prediction model is almost agnostic to the modality and scanner used. That is, except for the T2 weighted Gradient Echo MRIs obtained at one among 15 facilities, and that used the Signa HDxt GE Medical Systems 1.5 T scanner, we do not have to tell the model anything about the modality or scanner since the CNN was trained with all of them simultaneously and the best linear bias correction model is not moderated by modality or scanner type. This is a promising route for the development of useful brain age-based clinical tools that can be applied to clinical MRIs, which are known to manifest in a wide variety of contrasts due to the use of arbitrarily customized MR protocols (e.g., with varied Repetition Times, Echo Times, Inversion Times, slice thickness, averages) for specific clinical purposes.

The linear correction proved to be more useful in synthetic MPRAGEs, for which the MAE was worse than for the original MPRAGEs. When using the corrected model in the test sample, the MAE varied from 4.67–6.47 years across modalities. This is higher than that reported when using DeepBrainNet on research-grade MRI data (4.12 years)^2^, which is likely due to the use of the synthSR method and/or clinical MRI data. Also, the within-subject difference in absolute value of the corrected brain-PADs among modalities was not significantly different from zero, suggesting that all modalities performed relatively similarly. However, an interesting finding was that the use of SynthSR of actual MPRAGEs could yield to a better accuracy since MAE of the original MPRAGEs was 5.33 years and MAE of their synthetic counterparts was 4.67 years. This could owe to the fact SynthSR tends to improve contrast to noise ratio (e.g., compare them in Fig. 1) and may remove some artifacts of no biological origin.

Our MAE and linear bias was higher than that reported by Wood et al. (2022)^16^, where brain age was predicted from clinical T2w and DWI MRIs with a MAE < 4 years. This might be due to differences in the performance of the CNN architectures. For example, contrary to their study that used a 3D CNN, DeepBrainNet treats the 2D slices as independent samples and not the whole brain is covered. In addition, DeepBrainNet relies on preprocessing steps that could introduce errors in the prediction. For example, an incorrect brain extraction in the native space yields to both incorrect brain tissue classification and aberrant shearing when normalizing to the MNI template. But more importantly, we trained the model in a sample for which we do not have detailed clinical information. Even though we removed subjects with significant structural abnormalities, it is possible that a subsample of “non-healthy” participants could still be part of our dataset, affecting what the model considers a “healthy brain” for a certain age or leading to higher errors when testing. Moreover, as illustrated in Fig. 3, we were unable to eliminate all poorer quality MRIs, which influenced training and testing.

We suspect that, among the abovementioned possible causes, DeepBrainNet itself is the most likely culprit of the linear bias observed in our predictions. Indeed, a recent paper compared several deep learning methods to predict brain age using research-grade MRIs^22^. Figure 1 of their paper shows that DeepBrainNet yielded a linear bias more pronounced than other 3D deep learning models. Moreover, using clinical MRIs after retraining does not seem to considerably worsen the bias. Based on our analysis of the linear fit between brain age obtained using DeepBrainNet and chronological age plotted that figure (they do not directly report the value), we roughly estimate a slope of ~ 0.75, which is only slightly higher than the slope of 0.7 for our clinical MRIs. In any case, our approach is proof-of-concept for the use of super-resolution methods to develop modality and scanner-agnostic brain age prediction methods.

Finally, as hypothesized, the predictions were consistent across modalities and/or repetitions at the subject level (redundant according to the Cronbach’s alpha coefficients when excluding the T2wGREs), and the average within-subject variability in brain-PAD (the average within-subject MAE) was 2.4 years when excluding the T2wGRs, and 2 years when excluding all MRI modalities that were not used for training.

This study comes with several limitations. Although it was possible to predict brain age from multimodal MRIs, given we did not have enough information about the clinical characteristics of the patients, we were not able to determine how much of their possible underlying conditions are affecting the estimations. In fact, we did not test whether brain-PAD obtained from a clinical brain MRI is sensitive enough to underlying conditions (dementia, chronic pain, etc.). This should be the next study, provided we can gather enough clinical data from the patients. Also, chronological age was not evenly distributed in our sample. This could have biased model selection towards a model that best corrects brain ages of participants with more frequent chronological ages in the sample. However, including weights based on the inverse of the frequency the occurrence of the chronological ages (bestowing more importance to the lowest and highest ends of the chronological age distribution) did not improve accuracy during cross-validation.

The main limitation is that our model failed to accurately predict brain age from the T2wGREs obtained with the Signa HDxt GE 1.5 T at one facility. One of reasons for this failure could be that this modality was not part of the training set, and that would indicate the model needs to be trained with MRIs from a more varied pool of clinical MR protocols. Other causes could be related to some specific characteristics (e.g., technical, staff, personal data handlings) of that particular facility that could have affected the results beyond reasons related to the modality. This seems to be unlikely though, since the brain age prediction accuracy does not seem to be significantly affected when predicted from MRIs of other modalities also obtained at that facility. Bigger datasets with more diverse clinical MR protocols are needed to shed more light into this.

Finally, the performance of the model was likely affected by the fact we could not visually inspect all MRIs in the database. First, some MRIs with very poor quality (e.g., noise or artifacts) could have affected training and/or accuracy on the testing set. Likewise, the preprocessing steps could have had the same type of impact in both training and validation. Future study should avoid improper brain extraction to affect the affine normalization by introducing aberrant shearing. A viable route could be to first normalize the whole-head MRI and initialize the brain-extraction algorithm using the FSL’s template brain mask or adopt novel deep learning-based techniques for brain extraction like HD-BET^23^.

In summary, it is feasible to accurately predict brain age from any clinical MRIs from patients that visit the UF’s Health System in FL, USA. Future studies are needed to test the generalizability of these predictions to any clinical facility and to investigate the ability of the predicted brain age difference in multimodal clinical data to characterize pathological conditions. We stress that it is in clinical settings where brain age biomarkers (and any biomarker in general) are needed the most. We have demonstrated the feasibility of brain age predictions on clinical populations, taking additional steps toward the development of biomarkers in personalized medicine.

## Methods

All participants, or their legal guardians, gave informed consent for their clinical data to be used for research purposes. All methods were performed in accordance with the relevant guidelines and regulations. This research was performed in accordance with the declaration of Helsinki. MRI acquisition was carried out after all participants completed a screening form to determine MRI eligibility. We received de-identified MRI data. This study was approved by the Institutional Review Board of the University of Florida (IRB No. 202101469).

### DeepBrainNet architectures for brain age prediction

A DeepBrainNet consists of a CNN connected to a dense layer with 1024 units with a Rectified Linear Unit (ReLU) activation, an 80% dropout layer (when training), and a single output layer with linear activation (the predicted brain age)^2^. The CNN can be the “InceptionResNetV2”^19^ or the “Visual Geometry Group” network with 16 layers (VGG16)^20^, initialized with ImageNet weights before trained with MRI data^2^. Since these CNNs predict from 2D images, DeepBrainNet operates with the 2D slices of an MRI as independent samples. The individual’s predicted brain age is the median of the predictions across a set of slices. DeepBrainNet architectures were trained with preprocessed research-grade T1w images from 11,729 individuals (ages 3–95 years) from various geographic locations, scanners, acquisition protocols, and studies, and tested in an independent sample of 2,739 individuals. The required preprocessing for a T1w brain MRI is described in the next section.

### Preprocessing of the MRIs

We preprocessed all original and synthetic MPRAGEs as required for the use of DeepBrainNet. We skull-stripped them using *smriprep* (https://www.nipreps.org/smriprep/usage.html), i.e., the image was corrected for intensity non-uniformity using *N4BiasFieldCorrection*^24^ distributed with ANTs 2.2.0 and skull-stripped with a Nipype implementation of the *antsBrainExtraction.sh* workflow from ANTs^25^, using OASIS30ANTs as target template. Finally, we transformed the skull-stripped images to the 1-mm isotropic voxel FSL skull-stripped T1w template [“MNI152, LPS orientation (Right ◊ Left, Anterior ◊ Posterior, Inferior ◊ Superior), and 218 x 182 x 218 dimensions] using a 12-parameter linear affine transformation estimated via *spm_coreg.m* from the Statistical Parametric Mapping (SPM; https://www.fil.ion.ucl.ac.uk/spm/) and scaled them from 0 to 255. Finally, image intensities were scaled from 0 to 255.

The 2D images used by DeepBrainNet for prediction were the 80 slices centered at the z = 0 plane in MNI coordinates of the normalized T1w. Note that our chosen brain age prediction (i.e., DeepBrainNet) method does not rely on the whole-brain MRI. This is at least convenient for axial clinical-grade MRIs, since they often lack their topmost and bottommost slices.

### Quality control of the preprocessed MRIs

Preprocessed MRI images were submitted to a careful quality control (QC) procedure. Since the study included a large number of MRIs, only a subset of the preprocessed MRIs that were likely to have bad quality were visually inspected. The selection of this subset of MRIs was carried out as follows. We calculated the normalized mutual information (NMI)^26^ between the preprocessed MRIs and the 1-mm isotropic voxel FSL skull-stripped T1w template. We then plotted the histogram of the NMIs and visually defined a threshold based on those values appearing to be significantly below the main unimodal distribution. We inspected all images below this threshold and those above it until they had no visible preprocessing errors. Since the goal is to demonstrate feasibility of the brain age estimation for clinical images, which have generally less quality than those intended for research purposes, we were lenient regarding the consideration of what a “processing error” was. We only removed preprocessed MRIs that were indisputably unrecognizable due to motion, the brain extraction did not remove significant non-brain tissues, or the normalization performed poorly. In addition, we discarded images with significant structural abnormalities (e.g., tumors, deformations, very large ventricles, tissue loss).

### Data subdivisions

**Figure 5:**
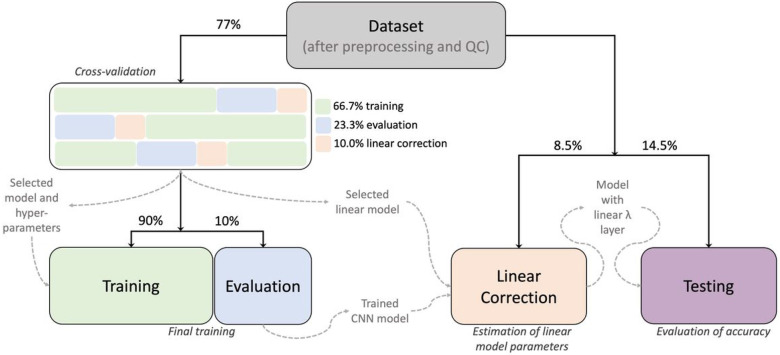
Flowchart of the definition of the data domains and the different stages of training and evaluation. After selecting the whole brain MRIs, preprocessing, and performing QC, the final dataset was split for training the CNN model (training set), estimating the parameters of model of the linear bias correction in a different independent sample (the linear correction set) and testing accuracy (external validation in the held-out testing set) and reliability. In more detail, three-fold cross-validation was used on the training set to determine the optimal combination of CNN model, tune hyperparameters and select a linear model. The final training was performed on the training set using the optimal configuration. The trained CNN model was then used to predict the uncorrected brain ages that, together with the chronological ages, were used to estimate the parameters of the linear bias in the independent linear correction set. Finally, the linear model was added on top of the CNN for deployment and tested in the held-out testing set.

Figure 5 depicts how the dataset was split for training and evaluations. Splits were done at the participant level, i.e., if a participant belonged to a certain data proportion or subdivision, all the slices of all the modalities of that participant belonged in that proportion or subdivision. We split the whole dataset into three main independent subdivisions: 77% for training, 8.5% for characterizing and correcting the “regression dilution” bias (i.e., the linear correction set), and 14.5% held-out for external validation. These very specific proportions were the consequence of the distribution of modalities in the whole sample, as we will explain here and evidence in the results methods. First, there were 4 modalities that had too few samples to be part of the training set. Thus, we decided that they could only be part of a sample used to test the predictions, specifically to test the accuracy in modalities that were not used to training the model. These four modalities were only in 14.5% of the participants, hence this proportion for the testing set. The training set was selected to be 70% plus its 10%, hence the total 77%, because 70% was used for the actual training and the rest was used to monitor accuracy and overfitting during the training process, as we explain in the next section. The remaining 8.5% of the sample was used to estimate the linear correction. Therefore, the training and linear correction sets had only MRIs among 5 different modalities, whereas the testing set had MRIs among the 8 modalities.

In addition, the training data were split into three folds for cross-validation of the models and hyperparameters. Following the same strategy previously described for the whole dataset, at each of the three iterations of cross-validation, two folds (i.e., 66.7% of the training data) were used for training and the remaining proportion was further subdivided: 30% for correcting the bias and 70% for evaluation (and to define early stopping) during training (10% and 23.3% of the whole training data, respectively).

Also, each subdivision had roughly the same distribution of the number of MRIs per participant, and this is why we could not use, for each iteration of the cross-validation, the same proportions for the training, linear correction, and evaluation we used for the whole dataset, as described above (i.e., 66.7%, 10% and 23.3% versus 77%, 8.5% and 14.5%, respectively). Finally, to ensure reproducibility, the samples were generated with a fixed seed at the beginning of the study.

### Re-training of the DeepBrainNet models

We used Keras 2.11 with the mean squared error (MSE) as the loss function, the mean absolute error (MAE) as the accuracy metric and the Adam optimizer^27^. To tune model hyperparameters, we cross-validated across a grid defined by the Cartesian product of the models (InceptionResnetV2 and VGG16) and the following values of the hyperparameters: learning rate = [7e-6, 7e-5, 7e-4], the batch size = [80, 160, 240, 320, 400] and a Boolean variable specifying whether weights of importance of the observations were applied during training–weights were inversely proportional to the frequency of occurrence of a certain chronological age range (defined by the bins of the histogram of the ages)–to avoid the algorithm to learn to predict brain ages more accurately for ages more frequent in the training data. The training data was shuffled at the slice level so a batch could include slices from several subjects.

For each cross-validation iteration and grid cell, we trained the original CNN model on the training subset, evaluated its performance using MAE on the validation subset, corrected the linear bias using the linear correction subset, and calculated a corrected MAE again in the validation subset. This was done as follows. We loaded the corresponding original DeepBrainNet model and, at first, set the weights of the fully connected and output layers as trainable, while the weights of the rest of the network were frozen. We trained this model using a maximum of 20 epochs. We then unfroze all the layers of the model and re-trained it with another maximum of 20 epochs. Early stopping was applied during training if there was no improvement of the loss function during the last three consecutive epochs (patience = 3) or if the MAE of the training data was lower than that of the validation data (patience = 0) to avoid overfitting. During training, we adjusted the learning rate after each batch using learningrate (batch)=initiallearningrate/ (1 + batch×decay), decay = 3e-7. After the end of the training, we used the trained model to predict the brain age of each slice of the linear correction subset of the cycle and computed the median within subjects to obtain the individual whole-brain brain ages. We then fitted the linear correction model, i.e., brainage∼prediction (in Wilkinson’s notation) in this subset to characterize the linear bias, where:

1. prediction=chronologicalage,
2. $prediction=chronological\;age{\;}^{*}\;modality$,
3. $prediction=chronological\;age{\;}^{*}\;scanner$, or
4. $prediction=chronological\;age{\;}^{*}\;modality{\;}^{*}\;scanner$.

We then added an additional layer to the model’s architecture (using the Keras’ “Lambda” layer) that takes both the predicted brain age and the chronological age as inputs and outputs a corrected brain age according to the formula: correctedbrain=age=chronologicalage+brainage−prediction. We shall call this new model the “corrected model” Finally, we predicted the brain age of the evaluation subset of the cycle using the trained uncorrected model to obtain an “uncorrected MAE” for that cycle and grid cell, and the corresponding corrected models to obtain four “corrected MAEs” for that cycle and grid cell.

We selected the models and training parameters that yielded the lowest MAE averaged across folds. We used the uncorrected MAE to select the CNN and set of training hyper-parameters. We then used the four corrected MAEs of the selected CNN/hyper-parameter combination (grid cell) to select the bias correction model. Using this final combination, we re-trained the original CNN model as described above, keeping only 10% whole training set to monitor early stopping and using the remaining 90% for training (hence the 77% to make the actual training set the typically used 70% in the literature). We then used the held-out linear correction set to correct the linear bias using the selected linear model. Finally, we evaluate the generalization error and internal consistency in the held-out testing set as described in the next section.

### Evaluation of the accuracy in the testing dataset (generalization error)

We reported the accuracy of the predictions using the sample MAE, the correlation between the predicted brain age and the chronological age, and the coefficient of determination ($R^{2}$) of the brainage=chronologicalage+error linear model (i.e., slope=1 and intercept=0). That is, with 〈x〉 being the average of x:

$$\;R^{2}=1-\left(\frac{RSS}{TSS}\right)\;RSS=\sum_{i}(brainage_{i}-chronologicalage_{i}{)}^{2}\;\;TSS=\sum_{i}(brainage_{i}-\langle brainage\rangle {)}^{2}$$

A perfect fit yields $R^{2}=1$. Also, with this constrained definition, $R^{2}$ can have a negative value when the model brainage=chronologicalage+error does not follow the trend of the data.

We adopted a non-parametric approach to calculate these measures of accuracy to deal with possible inflation due to repeated observations. We calculated these measures separately for each modality using a randomly selected repetition per subject. Since this method yields a different result for each run, we used bootstrapping to calculate the accuracy measures and took the average. Bootstrapping also allowed us to estimate the 95% confidence intervals (CIs). We used 10,000 × N_r_ bootstraps to account for the fact a random image among a maximum of N_r_ repeated measures is drawn for each subject and modality. When reporting the accuracy measures for the total sample, i.e., pooling from modalities and repetitions, we used 10,000 × N_r_ × N_m_ bootstraps, where N_m_ is the number of modalities.

To test part of our first hypothesis, that involves comparing predictive accuracies, we fitted the linear mixed model “∣brain-PAD∣∼modality+subject+(1∣subject)”, where ∣∣ takes the absolute value, *modality* is a categorical variable with a level for each modality, including the original MPRAGE. Note that this is a mixed effects repeated measures ANOVA with missing values in long format, i.e., $|brain-PAD_{i,r,m}|=s_{i}+\beta_{m}+\eta_{i}+e_{i,r,m}$ where, $s_{i}$ is the $ith^{’}s$ subject fixed effect, $\beta_{m}$ is the fixed effect of the mth modality, $\eta_{i}$ is $ith^{’}s$ subject the random effect and $e_{i,r,m}$ is a random error for theith, subject rth repetition and mth modality. The fit was done by maximizing the Restricted Maximum Likelihood using the ‘Quasi-Newton’ optimizer and modeling the covariance matrix using a full Cholesky parameterization. By modeling a non-diagonal covariance matrix and adding a random effect in the intercept across subjects, we are accounting for possible correlations between observations due to the use of repeated measures for some subjects (between and within modalities). This should avoid inflation of Type I errors when testing the significance of certain contrasts in the model. We then tested the within-subject comparison of the ∣brain-PAD∣ between the mth synthetic modality and the original MPRAGE by evaluating and testing the contrast that compared their corresponding coefficients in the model, i.e., $\beta_{m}-\beta_{MPRAGE}$,$\beta_{m}$ is the coefficient of the fixed effect of the mth modality.

Finally, note that this linear mixed model also provides an estimation of the absolute value of the brain-PAD of each subject’s modality given by $s_{i}+\beta_{m}$, where $s_{i}$ is the coefficient of the fixed effect of the ith subject. Thus, we can also report a “population” estimate of the MAE. To that end, we evaluated the contrast ${\hat{MAE}}_{m}=\langle s_{i}\rangle_{i}+\beta_{m}$, where $\langle \rangle_{i}$ denotes averaging across i, for an estimation of the MAE for each modality, and the contrast $\langle {\hat{MAE}}_{m}\rangle_{m}$ for an estimation of the total MAE.

### Reliability of brain age predictions across modalities and repetitions

To test our second hypothesis, we also evaluated the intra-subject reliability of the predictions, specifically, we evaluated Cronbach’s alpha on the brain-PAD. The Cronbach’s alpha is a measure of internal consistency ^21^. The ranges α ≥ 0.9 and 0.8 ≤ α < 0.9 denote an excellent and acceptable reliability, respectively, whereas lower values denote questionable, poor or even unacceptable reliability. Very high values of α ≥ 0.95 denote redundancy (which is desirable in our case). For the calculation of the Cronbach’s alpha, we reorganized the values into a matrix, X, of number of participants by modality/repetition pairs (the items). We considered all possible 6x4 = 24 modality/repetition pairs (i.e., the MPRAGE and the synthetic MRIs for all five modalities, and the maximum of four repetitions). We then dropped those participants (rows) having less than 3 items, and those items having more than 95% of missing values. Cronbach’s alpha was calculated using the following formula ^28^:

$$cronbach=\frac{numberofitems}{numberofitems-1}\left[1-\frac{trace(C)}{\sum_{ij}C_{ij}}\right],$$

where C is the covariance matrix of X. To handle the remaining missing values, C was calculated using pairwise elimination. The lower and upper bounds of a CI (e.g., 95%) for the Cronbach’s alpha were given by:

$$\;lower=1-(1-cronbach)\;F^{-1}\;(\alpha ∕2,df_{1},df_{2})\;upper=1-(1-cronbach)\;F^{-1}\;(1-\alpha ∕2,,df_{1},df_{2}){\;}^{’}$$

where $F^{-1}\;()$ is the inverse of the complementary cumulative distribution function (or inverse survival function) for the F-distribution, α=1−CI∕100,df1=numberofobservations−1, and df2=df1×numberofitems

## Figures and Tables

**Table 1:** Distribution of MRIs that passed QC among modalities and scanners.

Modality	Scanner									Number of repetitions			
	Aera	Avanto	Prisma	Sola	Signa HDxt	Skyra	Titan 3T	Verio	TOTAL	1	2	3	4
**MPRAGE**	148	182	162	45	73	25	30	182	**847**	833	7	0	0
**2D T1w**	268	518	240	38	154	32	115	438	**1803**	680	541	11	2
**2D T2w**	394	565	301	49	271	49	128	465	**2222**	606	763	26	3
**2D T1wFLAIR**	2	8	2	0	73	1	0	4	**90**	50	20	0	0
**2D T2wFLAIR**	234	120	227	48	158	40	82	108	**1017**	981	18	0	0
**2D T2wGRE**	0	0	0	0	151	0	0	0	**151**	151	0	0	0
**2D IR**	4	12	36	1	0	15	0	26	**94**	32	31	0	0
**TOTAL**	**1050**	**1405**	**968**	**181**	**880**	**162**	**355**	**1223**	**6224**	**3333**	**1380**	**37**	**5**

Note. The first section shows the number of MRIs per modality and scanner. The second section shows the number of subjects having 1, 2, 3 or 4 repetitions of each modality.

**Table 2:** Measures of accuracy in the brain age prediction using the selected retrained CNN (VGG16-based DeepBrainNet) for each modality in the testing held-out sample.

Modality	Testing Sample MAE		Correlation		$R^{2}$		Within-subject Difference with original MPRAGE		$\hat{\text{MAE}}$	
	Mean	95% CI	Mean	95% CI	Mean	95% CI	Mean	95% CI	Mean	95% CI
**MPRAGE**	5.31	[4.58, 6.19]	0.92	[0.88, 0.94]	0.79	[0.69, 0.86]			5.21	[4.03, 6.40]
**Synthetic MRIs**										
**MPRAGE**	6.43	[5.61, 7.41]	0.89	[0.84, 0.92]	0.60	[0.46, 0.71]	1.29	[−0.0, 2.57]	6.50	[5.34, 7.66]
**T1w**	8.07	[6.58, 9.00]	0.86	[0.80, 0.90]	0.28	[−0.14, 0.46]	3.26	[1.89, 4.62]	8.47	[7.34, 9.60]
**T2w**	6.78	[6.12, 7.54]	0.89	[0.85, 0.91]	0.60	[0.46, 0.67]	1.56	[0.44, 2.67]	6.77	[5.87, 7.67]
T1wFLAIR	7.55	[6.18, 8.90]	0.92	[0.88, 0.94]	0.45	[0.23, 0.65]	3.14	[1.59, 4.68]	8.35	[6.95, 9.75]
**T2wFLAIR**	6.19	[5.57, 6.96]	0.91	[0.88, 0.93]	0.71	[0.63, 0.78]	0.96	[−0.25, 2.17]	6.17	[5.15, 7.19]
IR	6.75	[5.26, 7.91]	0.83	[0.71, 0.89]	0.57	[0.36, 0.76]	1.1	[−0.41, 2.6]	6.31	[4.89, 7.73]
T2wGRE	**18.32**	**[16.52, 20.05]**	**0.79**	**[0.73, 0.83]**	**−5.09**	**[−6.87, −3.29]**	**13.08**	**[11.75, 14.4]**	**18.29**	**[17.16, 19.42]**
**All synthetic MRIs except T2wGRE**	6.90	[6.26, 7.75]	0.88	[0.85, 0.91]	0.58	[0.52, 0.72]	3.48	[2.47, 4.49]	7.09	[6.30, 7.89]

Note. The first, second and third columns are the bootstrapped MAE, Correlation and coefficient of determination of the brainage=chronologicalage linear model, respectively. The fourth column is the estimated within-subject difference in the corrected brain-PAD between each modality and the original MPRAGE. The fifth column is the “population” estimated MAE. MAE: Mean absolute error (in years). MPRAGE: Magnetization-prepared rapid gradient-echo. T1w: T1-weighted. T2w: T2-weighted. FLAIR: Fluid attenuated inversion recovery. GRE: Gradient Echo. IR: Inversion Recovery. T2wGRE results are highlighted in bold font and modalities that were not part of the training set are underlined.

**Table 3:** Measures of accuracy in the brain age prediction using the selected retrained CNN (VGG16-based DeepBrainNet) with the linear correction layer for the MRIs for each modality in the testing held-out sample.

Modality	Testing Sample MAE		Correlation		$R^{2}$		Within-subject Difference with original MPRAGE		$\hat{\text{MAE}}$	
	Mean	95% CI	Mean	95% CI	Mean	95% CI	Mean	95% CI	Mean	95% CI
**MPRAGE**	5.33	[4.70, 6.03]	0.96	[0.93, 0.97]	0.90	[0.87, 0.93]			5.28	[4.42, 6.14]
**Synthetic MRIs**										
**MPRAGE**	4.67	[4.10, 5.41]	0.95	[0.92, 0.96]	0.89	[0.84, 0.92]	−0.57	[−1.5, 0.36]	4.71	[3.87, 5.56]
**T1w**	5.74	[4.50, 6.03]	0.93	[0.91, 0.95]	0.83	[0.75, 0.88]	0.57	[−0.41, 1.56]	5.86	[5.04, 6.68]
**T2w**	4.99	[4.48, 5.54]	0.94	[0.92, 0.96]	0.89	[0.85, 0.91]	−0.33	[−1.14, 0.48]	4.95	[4.30, 5.60]
T1wFLAIR	4.81	[3.95, 5.69]	0.96	[0.94, 0.97]	0.89	[0.85, 0.93]	0.14	[−0.98, 1.26]	5.42	[4.40, 6.44]
**T2wFLAIR**	5.05	[4.59, 5.67]	0.95	[0.94, 0.96]	0.90	[0.87, 0.92]	−0.17	[−1.05, 0.71]	5.12	[4.38, 5.85]
IR	6.47	[5.61, 7.75]	0.91	[0.83, 0.94]	0.81	[0.72, 0.88]	0.91	[−0.19, 2.00]	6.19	[5.16, 7.22]
T2wGRE	**18.42**	**[17.18, 19.66]**	**0.93**	**[0.90, 0.94]**	**−0.99**	**[−1.51, −0.50]**	**13.31**	**[12.35, 14.27]**	**18.59**	**[17.77, 19.41]**
**All synthetic MRIs except T2wGRE**	5.21	[4.86, 5.94]	0.94	[0.92, 0.95]	0.88	[0.84, 0.91]	1.98	[1.25, 2.71]	5.37	[4.80, 5.95]

Note. The first, second and third columns are the bootstrapped MAE, Correlation and coefficient of determination of the correctedbrainage=chronologicalage linear model, respectively. The fourth column is the estimated within-subject difference in the corrected brain-PAD between each modality and the original MPRAGE. The fifth column is the “population” estimated MAE.

## Data Availability

The datasets generated and/or analyzed during the current study are not publicly available because the related project has not concluded, but are available from the corresponding author on reasonable request.
